# Integrative Analysis Reveals Genes Causal Relation with Ovarian Cancer and aging

**DOI:** 10.2174/0115680266358687250507041056

**Published:** 2025-05-09

**Authors:** Lan-hui Qin, Chongze Yang, Rui Song, Pei-yin Chen, Zijian Jiang, Weihui Xu, Guanzhen Zeng, Jin-yuan Liao, Liling Long

**Affiliations:** 1 Department of Radiology, Guangxi Medical University, No.22 Shuangyong Road, Nanning 530021, Guangxi, China;; 2 Department of Radiology, First Affiliated Hospital of Guangxi Medical University, No.6 Shuangyong Road, Nanning, Guangxi 530021, China

**Keywords:** Ovarian cancer, Aging, Summary data-based Mendelian randomization, Single-cell RNA sequencing, Genome-wide association study

## Abstract

**Background:**

Exploring the correlation between ovarian cancer and aging has great significance for understanding the pathogenesis of ovarian cancer and formulating targeted therapeutic regimens.

**Objective:**

This computational study aims to identify and validate key genes in monocyte subtypes related to ovarian cancer and aging, exploring potential causal relationships.

**Methods:**

We collected single-cell RNA sequencing data (GSE157007, GSE184880), GWAS data (14,049 samples and 40,941 controls from a European population), and eQTL data of ovarian cancer and aging. Using R software packages like Seurat and singleR, we conducted data integration, quality control, cell classification, and differential gene expression analysis to identify intersecting monocyte subtype genes in ovarian cancer and aging. We employed summary data-based Mendelian randomization (SMR) analysis and Heterogeneity in Dependent Instruments (HEIDI) tests to pinpoint causal genes. Further single-cell functional analyses (gene switching, cell communication, metabolic pathway analysis), Bulk RNA sequencing validation, functional enrichment, and protein-protein interaction (PPI) analyses elucidated these genes' biological roles.

**Results:**

The dataset included 123,280 cells, revealing differential gene expression in classical monocytes (104 genes), intermediate monocytes (43 genes), and myeloid dendritic cells (39 genes). SMR and HEIDI identified causal relationships for 7 genes in classical monocytes, 3 in intermediate monocytes, and 3 in myeloid dendritic cells with ovarian cancer. Bulk RNA seq validation confirmed six monocyte genes as causal in ovarian cancer and aging. TREM1, SERPINB2, and CD44 were upregulated, while DST was downregulated; SLC11A1 and PNRC1 showed contradictory patterns. Interactions with NK and T cells involved LGALS9 - CD44/45 receptors. Riboflavin metabolism was a common enriched pathway.

**Conclusion:**

This study identified six specific monocyte genes as potential therapeutic targets for ovarian cancer and aging.

## 1 INTRODUCTION

Ovarian cancer, a prevalent malignant tumor in gynecology, is frequently diagnosed in its advanced stages and is often referred to as the "silent killer." Additionally, ovarian cancer is highly malignant and resistant to the present platinum-based chemotherapy, resulting in extremely severe mortality and recurrence rates [1, 2]. Moreover, the availability of effective targeted treatment methods remains deficient [1, 2], which pose significant challenges in the management and treatment of ovarian cancer.

Aging is an inevitable process in human life, and studies have revealed a certain correlation between ovarian cancer and aging [3]. First, the risk of developing ovarian cancer increases with advancing age in women, suggesting a link between the aging process and the onset of ovarian cancer [4]. Second, some studies have indicated that the ovarian functional decline as the aging process may be related to the occurrence of ovarian cancer [5, 6]. Additionally, aging-related biological processes, such as reduced DNA repair mechanisms and increased chromosomal instability, may create favorable conditions for the occurrence of ovarian cancer [7]. Therefore, exploring the correlation between ovarian cancer and aging has great significance for understanding the pathogenesis of ovarian cancer and formulating targeted therapeutic regimens.

Single-cell sequencing is a powerful tool to identify the transcriptomic and epigenetic characteristics of different cell types in ovarian cancer tissues during aging, which can reveal cell heterogeneity and the presence of cell subpopulations [8-10]. This aids in understanding the dynamic processes of ovarian cancer and aging development to conduct potential drug targets and resistance mechanisms. Additionally, Summary-data-based Mendelian Randomization (SMR) analysis leverages genetic variants as instrumental variables to explore the causal relationships between specific genotypes in whole blood eQTLs and diseases [11, 12]. Across the research of ovarian cancer and aging, SMR analysis can help identify genetic variants related to ovarian cancer and aging to uncover new pathogenic mechanisms and therapeutic targets. Due to its contribution to exploring molecular mechanisms of ovarian cancer and aging, we also discovered new therapeutic targets, providing theoretical support for precision medicine and personalized treatment. The application of these advanced techniques brings new opportunities and challenges for scientific research and clinical practice in the fields related to ovarian cancer and aging.

To date, no studies have integrated single-cell RNA sequencing and SMR analysis studies on ovarian cancer and aging. To address this gap, we combined single-cell RNA sequencing data, GWAS datasets, and whole blood eQTL data. This integration aimed to identify genes with potential causal relationships to ovarian cancer and aging.

## 2 MATERIALS AND METHODS

### 2.1 Data Collection

We collected single-cell RNA sequencing data for 6 aging samples (GSM4750309_F020, GSM4750310_F021, GSM4750311_F023, GSM5684306_OH14, GSM5684307-OH15, GSM5684308_OH17) from GSE157007 [13]. The reason for eliminating the other three samples of the dataset is incomplete information. GSE184880 includes single-cell RNA sequencing data for 5 normal ovarian samples (GSM5599220- GSM5599224) and 7 ovarian cancer samples (GSM5599225- GSM5599231) [14]. Additionally, we collected GWAS data for ovarian cancer from IEU OpenGwas (https://gwas.mrcieu.ac.uk/), which includes 14,049 samples and 40,941 controls from a European population [15]. Subsequently, we collected peripheral blood eQTL summary data from the eQTLGen consortium (https://www.eqtlgen.org/cis-eqtls.html). Finally, we included a dataset of bulk RNA sequencing data for 19 ovarian cancer tissues (GSM4876147-GSM4876164) and 8 normal ovarian tissues (GSM4876140-GSM4876146) from GSE160626 (https://www.ncbi.nlm.nih.gov/geo/query/acc.cgi) for validation. This study utilized data from public databases, which are openly accessible and do not contain any identifiable personal or sensitive information. Consequently, according to our institution's policies and the terms of use for public databases, ethical review board (IRB) approval was not required for this research.

### 2.2 Single-cell RNA Sequencing Analysis

To integrate the single-cell RNA sequencing datasets of aging and ovarian cancer, we used the FindIntegrationAnchors function in Seurat with Reciprocal PCA (RPCA) as the parameter [16]. Concurrently, to ensure the quality of these datasets, we implemented stringent filtering criteria. These criteria included a threshold requiring more than two cells expressing a given gene, the number of genes expressed per cell being between 200 and 10,000, and mitochondrial gene expression being less than 10%. We then used the Seurat package for cell clustering with a resolution of 0.7. We utilized the singleR package to annotate automatic cell types, including seven main cell types: T cells, endothelial cells, monocytes, NK cells, tissue stem cells, B cells, and epithelial cells [17]. Four monocyte subtypes contain classical monocytes, intermediate monocytes, myeloid dendritic cells, and plasmacytoid dendritic cells. To assess whether the functions of monocyte subtypes are consistent between ovarian cancer and aging, we conducted cell communication analyses separately for each condition. To identify key genes of monocyte subtypes in ovarian cancer and aging, we applied the FindMarkers function to screen differentially expressed genes between a certain monocyte subgroup and other monocyte subgroups or other cells [18].

### 2.3 SMR Analysis

The key genes of monocyte subtypes in ovarian cancer and aging were extracted from the whole blood expression quantitative trait loci (eQTL) data. These genes, along with corresponding GWAS data, were analyzed using Summary Data-based Mendelian Randomization (SMR) analysis and Heterogeneity in Dependent Instruments (HEIDI) tests [19]. We used the default parameters of the SMR software (version 1.3.1). Genes with an SMR p-value < 0.05 and a HEIDI p-value > 0.05 were considered to have a causal relationship with ovarian cancer and aging.

### 2.4 Genes of Causality in Single-cell Functional Analysis and their Validation Through Bulk RNA Sequencing

We performed single-cell functional analysis on the genes causally related to ovarian cancer and aging. Initially, switch gene (driver gene) analysis was conducted on the integrated single-cell datasets of aging and ovarian cancer, illustrating the expression of switch genes over pseudo time using a timeline plot. Then, we employed scatter plots to display the expression of the causally related genes at different time points. Next, we analyzed the interactions between monocytes positive for each causally related gene and other cells, as well as their metabolic pathways. Finally, we validated the genes having a causal relationship with ovarian cancer and aging on the corresponding bulk RNA sequencing data [20].

### 2.5 Functional Enrichment Analysis and Protein-Protein Interaction (PPI) Analysis

To identify the enriched pathways of the corresponding genes, we performed functional enrichment analysis and Protein-Protein Interaction (PPI) analysis on the core genes obtained from the differential analysis of cell populations positive for each causally related gene using Metascape [21] (https://metascape.org/gp/index.html#/main/step1). We visualized the PPI results using Cytoscape (v3.7.0) [22].

To identify core gene interactions resulting from the PPI network, we typically preprocess the data to eliminate less relevant proteins, then calculate centrality metrics (such as degree and betweenness centrality) to rank nodes by their importance. Subsequently, dense modules are detected using clustering algorithms, allowing for the identification of tightly interacting protein groups, which are then integrated with functional enrichment analyses to confirm their biological significance.

### 2.6 Statistical Analysis

We performed R software (v4.2.1) and SMR software (v1.3.1) for data analysis. We conducted data analysis and visualization using R packages including Seurat, stringr, tidyverse, patchwork, reshape2, RColorBrewer, SingleCellExperiment, CellChat [23], viridis, mixtools, GeneSwitches, scMetabolism [24], rsvd, IOBR [25], data.table, ggplot2, among others.

In SMR analysis, a p-value less than 0.05 indicates a causal relationship between the gene and the disease. A b-value less than 0 suggests that the gene is a downregulated causal gene, whereas a b-value greater than 0 indicates an upregulated causal gene. HEIDI is a method used to detect false positives in SMR analysis. When HEIDI's p-value is less than 0.05, it indicates the presence of false positives, whereas a p-value greater than 0.05 suggests the accuracy and reliability of the SMR analysis results. Through switch gene analysis, a gene's R2 greater than 0 indicates it is an upregulated switch gene, while an R2 less than 0 indicates a downregulated switch gene.

## 3 RESULTS

### 3.1 Population Characteristics

**Fig. (1):**
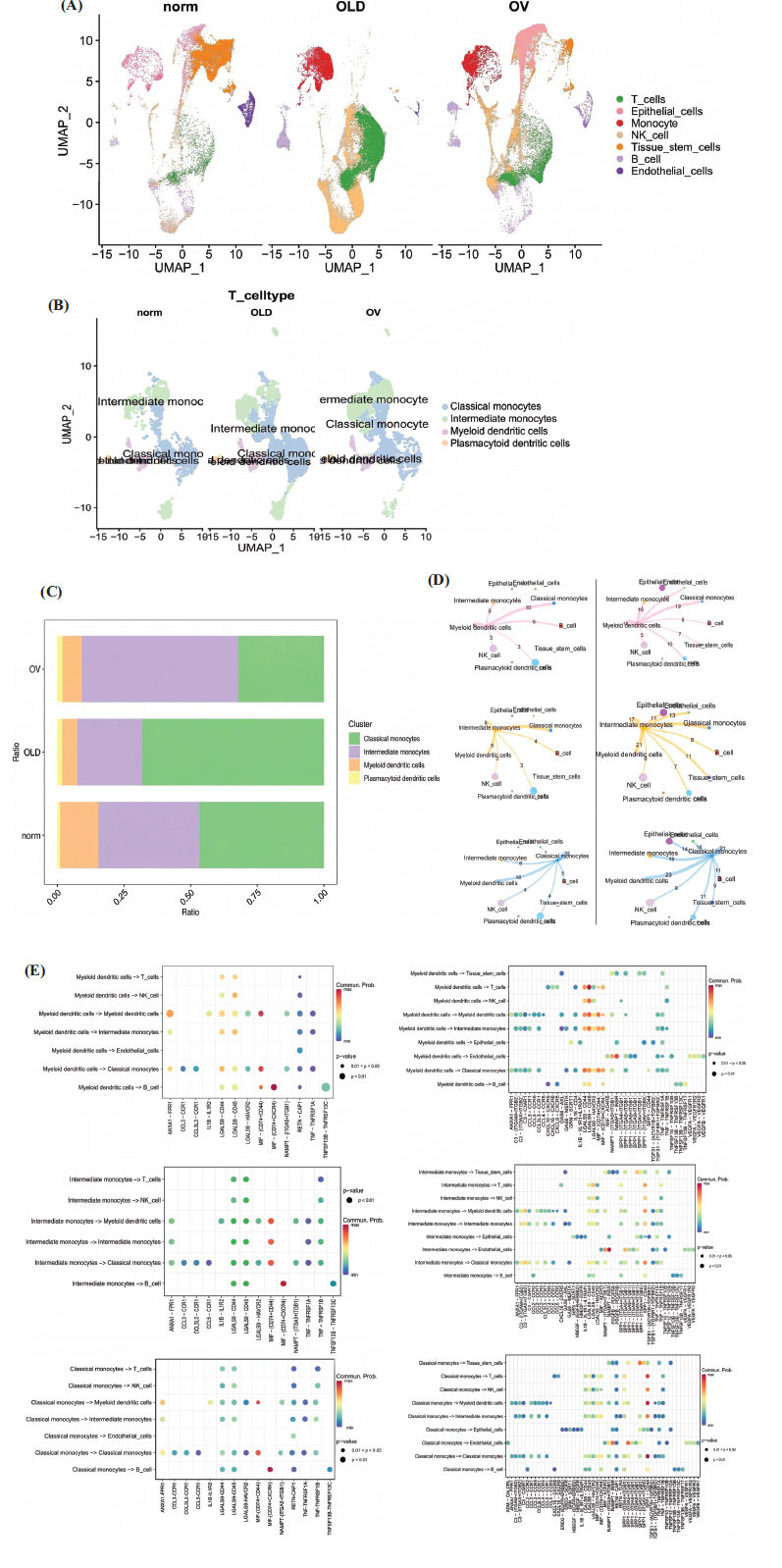
Integrated analysis of single-cell RNA sequencing datasets for ovarian cancer and aging. **A**) UMAP of cell types for each dataset; **B**) UMAP of monocyte subtypes for each dataset; **C**) Composition of monocyte subtypes in each dataset; **D**, **E**) Cell communication analysis of monocyte subtypes, with the left panel for the aging group and the right panel for the ovarian cancer group.

After rigorous filtering, the dataset in this study included 123,280 cells. Automatic cell type annotation was performed using a single R package. We obtained seven main cell types of T cells, endothelial cells, monocytes, NK cells, tissue stem cells, B cells, and epithelial cells, as well as four monocyte subtypes of classical monocytes, intermediate monocytes, myeloid dendritic cells, and plasmacytoid dendritic cells (as shown in Figs. **1A**-**C**). However, the number of plasmacytoid dendritic cells was small, at 266, which might be subject to sample bias and statistical instability. Consequently, we excluded them from further analysis.

We investigated cell-cell interactions using CellChat [23] analysis and discovered that the frequency and intensity of interactions between monocyte subtypes and other cells were higher in the ovarian cancer group compared to the aging group (Fig. **1D**). Notably, in both the aging and ovarian cancer groups, the interactions between classical monocytes, intermediate monocytes, myeloid dendritic cells, and their interactions with NK cells and T cells were mediated by ligand-receptor pairs such as LGALS9 - CD44/45, MIF - (CD74+CD44), and ANXA1-FPR1. However, the ligand-receptor pair SPP1-CD44 was only found in the ovarian cancer group. The cell-cell interaction relationships between classical monocytes, intermediate monocytes, myeloid dendritic cells, and other cells are shown in Fig. (**1E**).

### 3.2 SMR Analysis

**Fig. (2):**
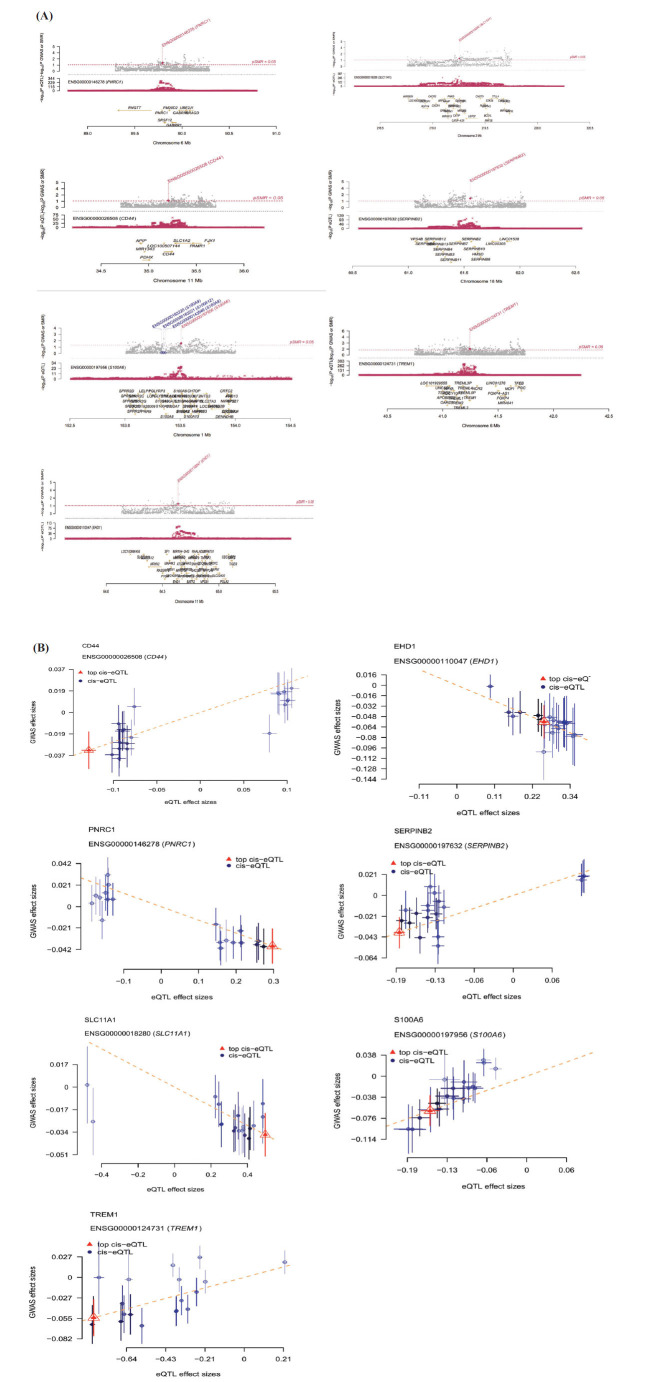
SMR analysis of classical monocyte genes with ovarian cancer based on GWAS. **A**) Genomic plots of classical monocyte genes; **B**) SMR causal effect plots for classical monocyte genes.

**Fig. (3):**
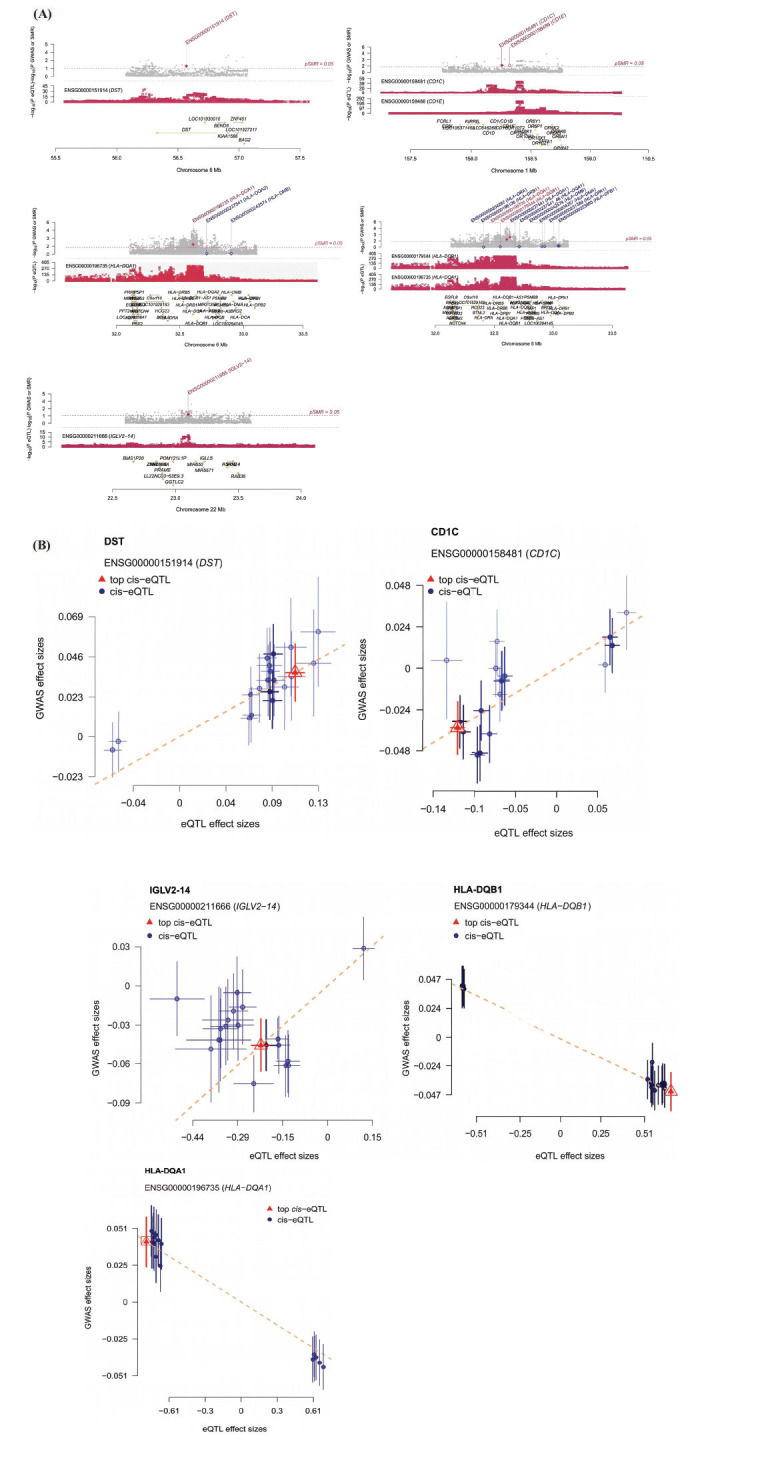
SMR analysis of intermediate monocyte and myeloid dendritic cell genes with ovarian cancer based on GWAS. **A**) Genomic plots for genes of intermediate monocytes and myeloid dendritic cells; **B**) SMR causal effect plots for intermediate monocytes and myeloid dendritic cell genes.

Following differential expression analysis among classical monocytes, intermediate monocytes, and myeloid dendritic cells, as well as between these cells and other cell types, we identified their common differential genes. These genes likely play a crucial role in defining the specific functions and characteristics of classical monocytes, intermediate monocytes, and myeloid dendritic cells. These genes include 104 genes in classical monocytes, 43 genes in intermediate monocytes, and 39 genes in myeloid dendritic cells. Subsequent SMR analysis and HEIDI tests on the meaningful data revealed that 7 genes in classical monocytes, 3 genes in intermediate monocytes, and 3 genes in myeloid dendritic cells have a causal relationship with ovarian cancer (as shown in Table **1**). Among them, CD44 and EHD1 in classical monocytes are both located on chromosome 11 (Fig. **2A**). In classical monocytes, CD44, S100A6, SERPINB2, and TREM1 genes had a positive causal relationship with ovarian cancer, while PNRC1, EHD1, and SLC11A1 had a negative causal relationship (Fig. **2B**). Intermediate monocytes and myeloid dendritic cells share the gene HLA-DRA1 on chromosome 6, which has a negative causal relationship with ovarian cancer (Fig. **3**).

### 3.3 Single-cell Functional Analysis

The expression of the causally related genes in various cell types was exhibited in Fig. (**4A**), and their expression in different monocyte subtypes was shown in Fig. (**4B**).

#### 3.3.1 Switch Gene Analysis

**Fig. (4):**
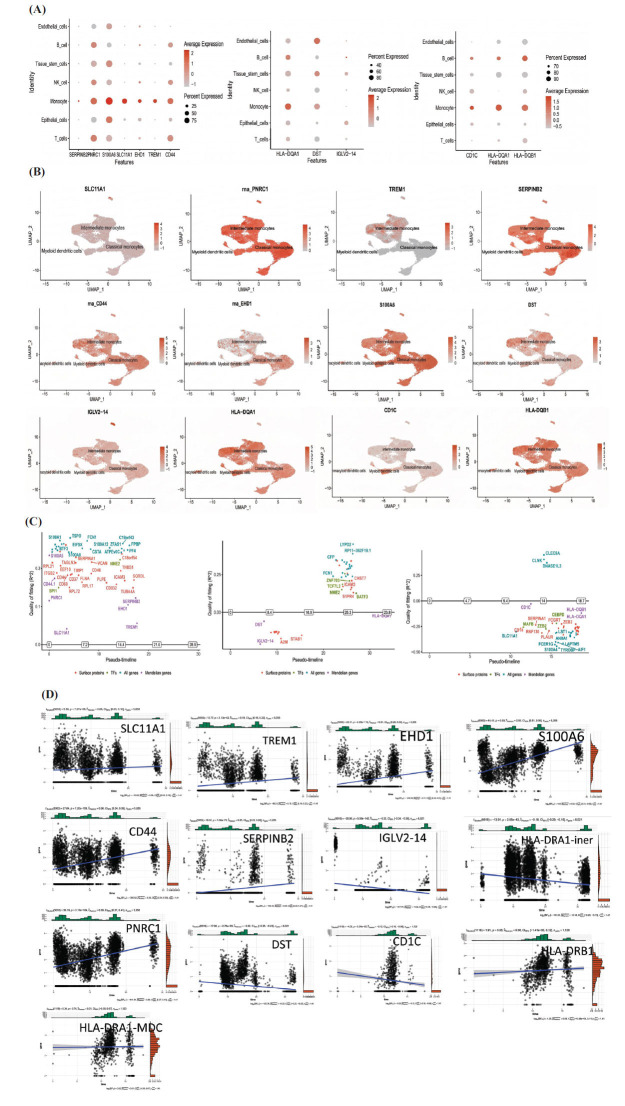
Expression and switch gene analysis of causally related genes in ovarian cancer and aging across different cell types. **A**) Expression of causally related genes across different cell types; **B**) Expression of causally related genes over pseudotime; **C**) Scatter plots showing the expression of causally related genes at different time points.

The switch gene analysis results indicate that all seven genes in classical monocytes are upregulated, whereas the genes in intermediate monocytes and myeloid dendritic cells are downregulated (Figs. **4C**, **D**). The highly expressed genes at the early stage of ovarian cancer and aging include PNRC1, S100A6, CD44, SLC11A1; while those expressed later are TREM1, EHD1, SERPINB2.

In the case of intermediate monocytes and myeloid dendritic cells in ovarian cancer and aging samples, we observed dysregulation of DST, IGLV2-14, and CD1C at early stages, while later stages found the dysregulation of HLA-DRA1 and HLA-DRB1.

#### 3.3.2 Cell Communication Analysis

**Fig. (5):**
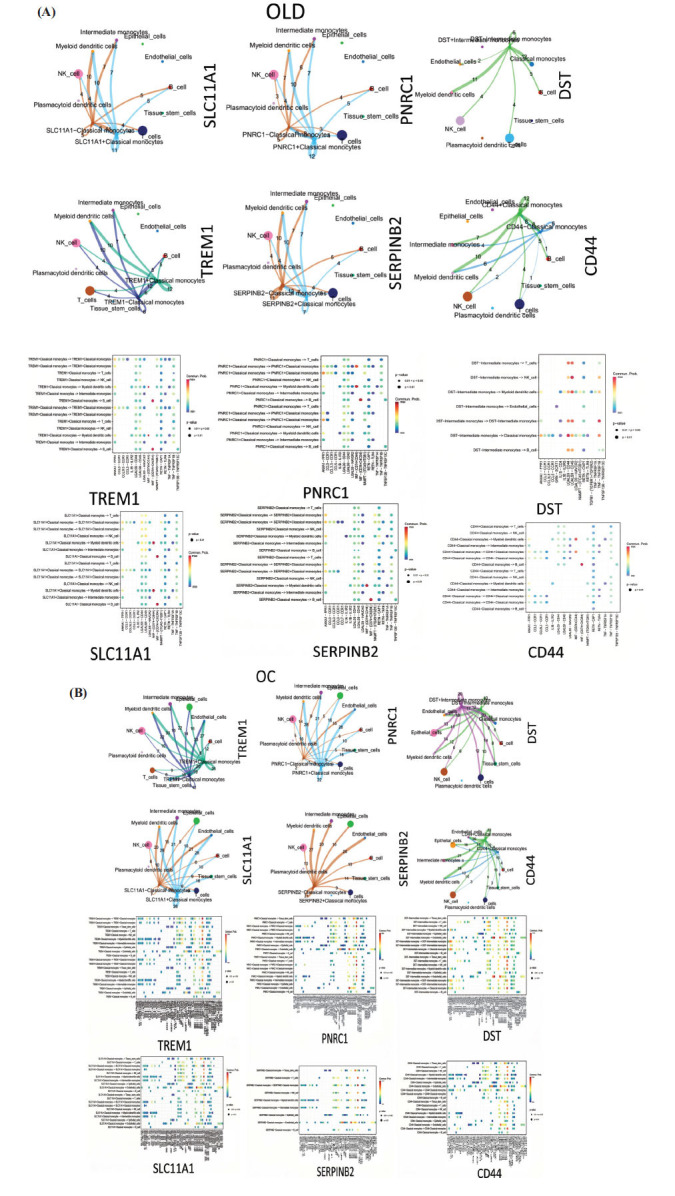
Cell-cell communication analysis of causally related gene-positive monocytes in ovarian cancer and aging. **A**) Cell-cell communication analysis of causally related gene-positive monocytes in ovarian cancer; **B**) Cell-cell communication analysis of causally related gene-positive monocytes in aging.

Our findings revealed that, compared to the aging group, the interactions between monocytes positive for causally related genes and other cells were both more frequent and intense in the ovarian cancer group (Figs. **5A**, **B**). Notably, in both the aging and ovarian cancer groups, ligand-receptor pairs such as LGALS9-CD44/45, MIF-(CD74+CD44), and ANXA1-FPR1 mediated the interactions between NK cells, T cells, and monocyte subtypes positive for causally related genes. However, the ligand-receptor pair SPP1-CD44 was only present in the ovarian cancer group. In the ovarian cancer group, the receptor-ligand pairs for causally related gene-positive monocytes and endothelial cells were exclusively NAMPT-INSR (Figs. **5A**, **B**, and Supplementary Figs. **1**, **2**).

#### 3.3.3 Metabolic Pathway Analysis

**Fig. (6):**
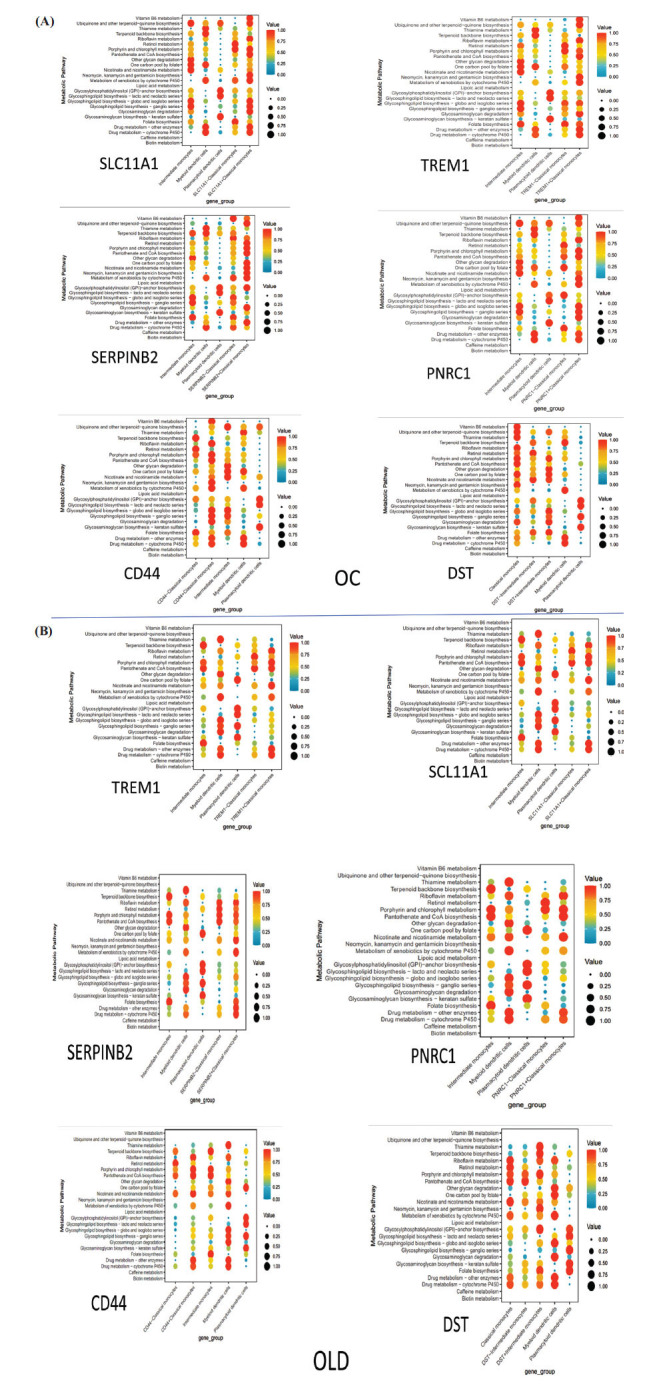
Metabolic pathways of causally related gene-positive monocytes in ovarian cancer and aging. **A**) Metabolic pathways of causally related gene-positive monocytes in ovarian cancer. **B**) Metabolic pathways of causally related gene-positive monocytes in aging.

In classical monocytes in aging and ovarian cancer, except for SERPINB2, the remaining six genes are commonly enriched in the Riboflavin metabolism pathway (Figs. **6A** and **B**). In classical monocytes, specifically in ovarian cancer, all genes except EHD1 and S100A6 are commonly enriched in metabolic pathways such as glycosaminoglycan degradation, neomycin, kanamycin, and gentamicin biosynthesis. Similarly, in myeloid dendritic cells in aging and ovarian cancer, three genes are commonly enriched in metabolic pathways, including Drug metabolism- cytochrome P450, Metabolism of xenobiotics by cytochrome P450, and other glycan degradation (Supplementary Fig. **3**). However, in intermediate monocytes, the positively identified genes did not share any common metabolic pathways.

### 3.4 Causal Gene Positive Monocytes Core Gene Enrichment Pathway

Fig. (**7A**) and Supplementary Fig. (**4A**) illustrate the functional enrichment pathways of core genes in monocytes positive for causally related genes. The common enrichment pathway for CD44, EHD1, PNRC1, SERPINB2-positive classical monocytes was positive regulation of response to external stimulus (GO:0032103). The common enrichment pathway for IGLV2-14 and DST-positive intermediate monocytes was Cytokine Signaling in Immune system (R-HSA-1280215). The common enrichment pathways for DST and HLA-DQA1 positive intermediate monocytes are hsa05150 Staphylococcus aureus infection and inflammatory response (GO:0006954), regulation of immune effector process. In myeloid dendritic cells positive for the three identified genes, the enriched pathways include positive regulation of immune response, inflammatory response, and cell activation.

### 3.5 Protein-protein Interaction Network (PPI Network)

**Fig. (7):**
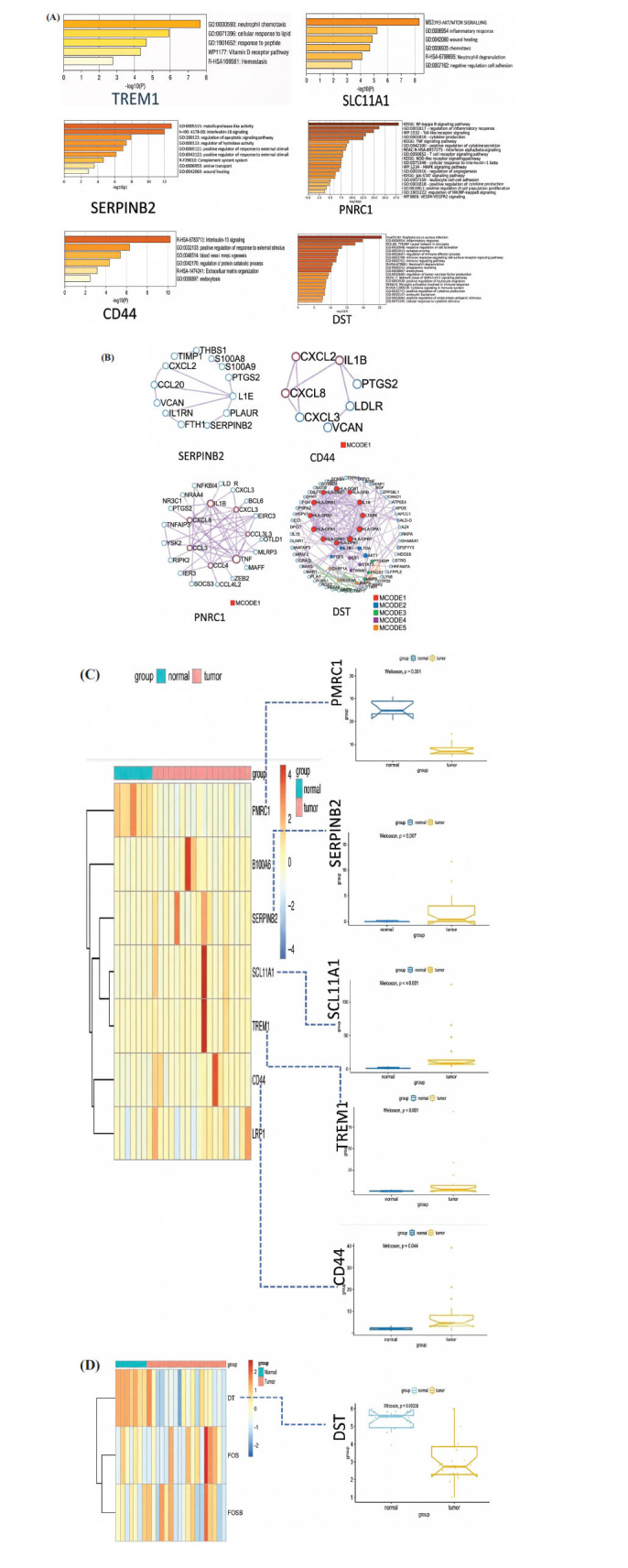
Analysis of core gene enrichment pathways, PPI, and validation of causally related gene-positive monocytes in ovarian cancer and aging. **A**) Core gene enrichment pathways in positive monocytes for causally related genes; **B**) Core gene PPI (Protein-Protein Interaction) in positive monocytes for causally related genes; **C** and **D**) Validation analysis of causally related genes in monocyte subtypes.

Fig. (**7B**), Supplementary Fig. (**4B**), and Tables **2**, **3**, and **4** depict the protein-protein interaction networks of key genes in monocytes positive for causally related genes. The core gene interactions of CD44 and PNRC1-positive classical monocytes are involved in the NF-kappa B signaling pathway (Fig. **7B** and Supplementary Fig. **4B**). The core gene interactions of CD44, EHD1, SERPINB2 positive classical monocytes are involved in the IL-17 signaling pathway and Interleukin-10 signaling. Meanwhile, the core gene interactions in classical monocytes positive for PNRC1, S100A6, and SERPINB2 are involved in the inflammatory response. The core gene interactions of DST, HLA-DQA1 positive intermediate monocytes, and CD1C, HLA-DRB1, HLA-DRA1 positive myeloid dendritic cells are involved in antigen processing and presentation of exogenous peptide antigen *via* MHC class II, antigen processing and presentation of peptide antigen *via* MHC class II.

### 3.6 Bulk Seq Validation

We validated causally related genes in ovarian cancer, and the bulk RNA sequencing results showed the expression of each gene in ovarian cancer and normal ovarian tissues (Figs. **7C**, **D**, and Supplementary Figs. **4C**, **D**). Among them, five genes in classical monocytes—namely, TREM1, SLC11A1, SERPINB2, PNRC1, CD44—exhibited statistical significance (Fig. **7C**). DST in intermediate monocytes also had statistical significance (Fig. **7D**). Except for PNRC1 and DST, which were downregulated, the rest were upregulated from these six significant genes.

## 4 DISCUSSION

To identify genes with potential causal relationships with ovarian cancer and aging, we employed novel bioinformatics approaches, including single-cell RNA sequencing analysis, genome-wide association studies (GWAS), and B Summary-data-based Mendelian Randomization (SMR). By integrating single-cell RNA sequencing data for ovarian cancer and aging, we identified intersecting differentially genes and conducted SMR analysis on these genes using GWAS and whole blood eQTL data. This analysis revealed 13 genes with causal relationships with ovarian cancer and aging. Subsequently, we performed single-cell functional analysis and bulk RNA sequencing validation on these 13 genes. Ultimately, we identified 6 genes with causal relationships. We identified TREM1, SLC11A1, SERPINB2, and CD44 as upregulated genes, while PNRC1 and DST were identified as negative regulatory genes.

We hypothesize that the high expression of TREM1, SERPINB2, CD44, and the dysregulation of DST may promote the onset of ovarian cancer and aging. We investigated that TREM1, SERPINB2, and CD44 were upregulated in SMR, switch gene analysis, and bulk RNA sequencing validation for ovarian cancer and aging, while DST was downregulated. First, upregulated TREM1, SERPINB2, and CD44 might be influenced by similar regulatory mechanisms [26-33] in both ovarian cancer and aging. This is caused by the function of common regulatory factors [34-43] such as transcription factors (*e.g*., NF-κB, AP-1, STAT3) and miRNAs (*e.g*., miR-155) in ovarian cancer and aging. Additionally, TREM1, SERPINB2, and CD44 may be involved in regulating signaling pathways, metabolic pathways, or cellular functions related to ovarian cancer and aging. Research has shown that TREM1 can promote tumor cell proliferation, invasion, and metastasis by activating the NF-κB signaling pathway [44], and is also involved in regulating tumor-related inflammatory responses. Moreover, previous research has indicated that TREM1 can participate in the aging process by activating the NF-κB signal pathway [45, 46]. Martincuks *et al*. demonstrated that the STAT3 transcription factor, mediated by the CD44 signaling pathway, promotes angiogenesis, immune suppression, and metabolic reprogramming in ovarian cancer [47]. From the metabolic level of classical monocytes, we found a common enrichment pathway of Riboflavin metabolism in both ovarian cancer and aging. Studies by Darguzyte M. *et al*., and Feng Y. *et al*., have confirmed that Riboflavin metabolism is involved in cell proliferation and angiogenesis in many tumors [48, 49]. We hypothesize that in ovarian cancer and aging, the various TREM1, SERPINB2, and CD44 positive monocyte subtypes interact with other cells through common ligand pairs, such as LGALS9-CD44/CD45. These interactions are involved in regulating biological processes, including mitochondrial autophagy and apoptosis, within the tumor microenvironment [50, 51]. These same receptor pairs and metabolic pathways might be derived from cross-regulatory situations, where they could interact through complex regulatory networks. However, although TREM1, SERPINB2, and CD44 are upregulated in both ovarian cancer and aging, there might be differences in their expression levels or regulatory mechanisms between ovarian cancer and aging. For instance, in ovarian cancer, the interaction receptor pairs for myeloid dendritic cells, intermediate monocytes, classical monocytes with endothelial cells are NAMPT-INSR, closely related to tumor energy metabolism and cell proliferation, a phenomenon not observed in aging [52, 53]. Similarly, from the metabolic level of monocytes, we found ovarian cancer-specific enrichment in pathways such as Glycosaminoglycan degradation, Neomycin, kanamycin, and gentamicin biosynthesis, Glycosaminoglycan degradation, which play crucial roles in maintaining tissue structure and function and regulating cell signaling with immune modulation. Meanwhile, we observed that DST is downregulated in both ovarian cancer and aging, suggesting that its dysregulation might contribute to the occurrence and progression of ovarian cancer [54]. However, the enriched pathways of DST at the metabolic level of monocytes in ovarian cancer and aging are different. In ovarian cancer, it is mainly enriched in Glycosphingolipid biosynthesis - globo and isoglobo series [55], potentially playing a role in cell recognition and adhesion. In aging, it is enriched in Neomycin, kanamycin, and gentamicin biosynthesis and Terpenoid backbone biosynthesis, which might regulate intracellular signaling pathways. In summary, the critical roles and regulatory mechanisms involving TREM1, SERPINB2, CD44, and DST in ovarian cancer and aging highlight their potential as therapeutic targets.

We observed that SLC11A1 and PNRC1 exhibit a paradoxical expression pattern across different analytical methods in ovarian cancer and aging, which may reflect their complex regulation and functional diversity in biological processes. Regarding SLC11A1, we noticed a downregulated trend in SMR analysis, yet it appeared upregulated in switch gene analysis and bulk RNA sequencing validation. This contrary expression pattern might indicate a dual regulation of SLC11A1 under different physiological states or environmental conditions. For instance, SMR analysis might capture the baseline expression level of SLC11A1[56], whereas switch gene analysis and bulk RNA sequencing validation could reflect the inducible expression of SLC11A1 in monocytes during ovarian cancer and aging [57]. Conversely, we observed that PNRC1 showed downregulation in SMR analysis and bulk RNA seq validation but upregulated in switch gene analysis. This is an intriguing phenomenon that may reflect the regulatory modes and biological functions of PNRC1 under different conditions. SMR analysis and bulk RNA sequencing validation provide insights into the expression changes of PNRC1 under knockout of the gene or at ovarian cancer states, consistent with conditions like cholangiocarcinoma and papillary renal cell carcinoma [58, 59], while switch gene analysis might reveal the expression response of PNRC1 in monocytes during ovarian cancer and aging under certain stress or specific signal pathway activation, with its expression also being elevated in COVID-19 [60]. The contradictory expression patterns of SLC11A1 and PNRC1 suggest complex regulation and multifunctionality in the processes of ovarian cancer and aging. These inconsistencies highlight the need for further research to uncover the specific functions and regulatory mechanisms of SLC11A1 and PNRC1. Such insights could reveal new therapeutic targets or strategies for the treatment of ovarian cancer and aging.

## STUDY LIMITATION

This article has certain limitations. First, although we have identified key genes, it is mainly focused on computationally analyzing previously published single-cell RNA sequencing data and other data retrieved from online datasets. Thus, we plan to carry out cytological and zoological experiments to validate our results, such as RT-qPCR, Western blotting, and a mouse model of ovarian cancer in follow-up studies. Second, this study only included monocytes and their three subtypes for analysis; in the future, we will extend our research to other immune cells or epithelial cells in the context of ovarian cancer and aging. Third, it is single-centered, which may affect the generalizability of the results. To enhance the robustness of our findings, we plan to conduct multicenter studies in the future.

## CONCLUSION

By integrating and analyzing single-cell RNA sequencing data, GWAS data, and whole blood eQTL data for ovarian cancer and aging, we identified six genes associated with ovarian cancer and aging, revealing potential causal relationships. In three monocyte subtypes, TREM1, SERPINB2, and CD44 were upregulated while DST was downregulated in the context of ovarian cancer and aging. We hypothesize that the various TREM1, SERPINB2, and CD44-positive monocyte subtypes interact with other cells through common ligand pairs, such as LGALS9-CD44/CD45. Intriguingly, we also discern that the enriched pathways of DST at the metabolic level of monocytes in ovarian cancer and aging are different. By elucidating the molecular mechanisms underlying these conditions, this research provides a foundation for the development of precision medicine approaches that could significantly improve patient outcomes. Further studies are needed to validate these targets and translate these findings into clinical applications, potentially revolutionizing the management and treatment of ovarian cancer and aging.

## Figures and Tables

**Table:** Results of SMR analysis for genes of different monocyte subtypes with ovarian cancer.

**-**	**probeID**	**ProbeChr**	**Gene**	**topSNP**	**A1**	**A2**	**Freq**	**b_SMR**	**se_SMR**	**p_SMR**	**p_HEIDI**
classic	-	-	-	-	-	-	-	-	-	-	-
-	ENSG00000197632	18	SERPINB2	rs6567401	A	G	0.477137	0.206458	0.085907	1.62E-02	1.36E-01
-	ENSG00000146278	6	PNRC1	rs10080656	T	C	0.324056	-0.1313	0.056731	2.06E-02	6.47E-01
-	ENSG00000197956	1	S100A6	rs2480695	G	T	0.091451	0.40581	0.180938	2.49E-02	2.57E-01
-	ENSG00000018280	2	SLC11A1	rs17229016	A	G	0.342942	-0.07276	0.032569	2.55E-02	6.47E-01
-	ENSG00000110047	11	EHD1	rs1211284	A	G	0.122266	-0.21792	0.098063	2.63E-02	9.24E-01
-	ENSG00000124731	6	TREM1	rs4714449	T	C	0.111332	0.06569	0.029712	2.70E-02	1.41E-01
-	ENSG00000026508	11	CD44	rs927335	T	A	0.346918	0.244429	0.119896	4.15E-02	3.72E-01
inter	-	-	-	-	-	-	-	-	-	-	-
-	ENSG00000196735	6	HLA-DQA1	rs1142323	G	A	0.300199	-0.05178	0.021231	1.47E-02	8.93E-01
-	ENSG00000151914	6	DST	rs6899968	C	A	0.351889	0.340283	0.152638	2.58E-02	8.26E-01
-	ENSG00000211666	22	IGLV2-14	rs34051623	T	C	0.333996	0.211726	0.098322	3.13E-02	1.80E-01
-	-	-	-	-	-	-	-	-	-	-	-
MDC	ENSG00000158481	1	CD1C	rs6699757	C	T	0.460239	0.300841	0.134624	2.54E-02	2.54E-01
-	ENSG00000196735	6	HLA-DQA1	rs1142323	G	A	0.300199	-0.05178	0.021231	1.47E-02	8.93E-01
-	ENSG00000179344	6	HLA-DQB1	rs9273493	T	C	0.428429	-0.06724	0.023464	4.16E-03	9.85E-01

**Note:** p_SMR < 0.05, p_HEIDI > 0.05 indicates a causal relationship between the gene and ovarian cancer. B_SMR represents the beta value from SMR analysis; 'classic' refers to classical monocytes, 'inter' refers to intermediate monocytes; MDS represents myeloid dendritic cells.

**Table 2:** Core gene PPI of positive classical monocytes for each causal relationship gene.

**Gene Name**	**Network/MCODE**	**GO**	**Description**	**Log10(P)**
CD44	Top3	-	-	-
-	-	hsa04657	IL-17 signaling pathway	-11.3
-	-	hsa04064	NF-kappa B signaling pathway	-11
-	-	WP2431	Spinal cord injury	-10.7
-	MCODE_1	WP3617	Photodynamic therapy induced NF kB survival signaling	-8.8
-	MCODE_1	R-HSA-6783783	Interleukin-10 signaling	-8.5
-	MCODE_1	hsa05134	Legionellosis	-8.2
EHD1	Top3	-	-	-
-	-	R-HSA-6783783	Interleukin-10 signaling	-11.3
-	-	hsa04657	IL-17 signaling pathway	-10.1
-	-	hsa04668	TNF signaling pathway	-9.7
PNRC1	Top3	-	-	-
-	-	hsa04064	NF-kappa B signaling pathway	-21
-	-	R-HSA-6783783	Interleukin-10 signaling	-16.9
-	-	GO:0006954	inflammatory response	-16.3
-	MCODE_1	R-HSA-6783783	Interleukin-10 signaling	-19.9
-	MCODE_1	WP5095	Overview of proinflammatory and profibrotic mediators	-16.7
-	MCODE_1	WP3624	Lung fibrosis	-15.3
S100A6	Top3	-	-	-
-	-	GO:0006954	inflammatory response	-40.2
-	-	M5885	NABA MATRISOME ASSOCIATED	-31
-	-	GO:0071621	granulocyte chemotaxis	-26.7
-	MCODE_1	R-HSA-418594	G alpha (i) signaling events	-23.8
-	MCODE_1	R-HSA-373076	Class A/1 (Rhodopsin-like receptors)	-23.6
-	MCODE_1	R-HSA-375276	Peptide ligand-binding receptors	-23
-	MCODE_2	GO:0030301	cholesterol transport	-11.1
-	MCODE_2	GO:0015918	sterol transport	-10.7
-	MCODE_2	GO:0034381	plasma lipoprotein particle clearance	-10.4
-	MCODE_3	R-HSA-166663	Initial triggering of complement	-15
-	MCODE_3	GO:0006956	complement activation	-12.9
-	MCODE_3	R-HSA-166658	Complement cascade	-12.9
-	MCODE_4	R-HSA-5668599	RHO GTPases Activate NADPH Oxidases	-12.5
-	MCODE_4	R-HSA-1236973	Cross-presentation of particulate exogenous antigens (phagosomes)	-10.3
-	MCODE_4	R-HSA-1236975	Antigen processing-Cross presentation	-9.9
-	MCODE_5	R-HSA-112409	RAF-independent MAPK1/3 activation	-8.8
-	MCODE_5	GO:0035335	peptidyl-tyrosine dephosphorylation	-8.4
-	MCODE_5	R-HSA-5675221	Negative regulation of MAPK pathway	-8
-	MCODE_6	R-HSA-2132295	MHC class II antigen presentation	-6.6
-	MCODE_6	hsa04210	Apoptosis	-6.5
-	MCODE_6	GO:2001235	positive regulation of apoptotic signaling pathway	-6.4
-	MCODE_7	M169	PID INTEGRIN2 PATHWAY	-9.1
-	MCODE_7	M159	PID AMB2 NEUTROPHILS PATHWAY	-8.6
-	MCODE_7	M174	PID UPA UPAR PATHWAY	-8.6
-	MCODE_8	R-HSA-2172127	DAP12 interactions	-8.5
-	MCODE_8	R-HSA-198933	Immunoregulatory interactions between a Lymphoid and a non-Lymphoid cell	-7.1
-	MCODE_8	R-HSA-1280218	Adaptive Immune System	-4.8
-	MCODE_9	WP2864	Apoptosis related network due to altered Notch3 in ovarian cancer	-8.3
-	MCODE_9	R-HSA-6785807	Interleukin-4 and Interleukin-13 signaling	-7.4
-	MCODE_9	R-HSA-449147	Signaling by Interleukins	-5.4
SERPINB2	Top3	-	-	-
-	-	R-HSA-6783783	Interleukin-10 signaling	-13.8
-	-	hsa04657	IL-17 signaling pathway	-11.9
-	-	GO:0006954	Inflammatory response	-10.8
SLC11A1	Top3	-	-	-
-	-	M53	PID INTEGRIN3 PATHWAY	-7.3
-	-	M18	PID INTEGRIN1 PATHWAY	-6.7
-	-	GO:0050769	Positive regulation of neurogenesis	-5

**Table 3:** Core gene PPI of positive intermediate monocytes for each causal relationship gene.

**Gene Name**	**Network/MCODE**	**GO**	**Description**	**Log10(P)**
DST	Top3	-	-	-
-	-	hsa05150	Staphylococcus aureus infection	-28.6
-	-	GO:0002478	antigen processing and presentation of exogenous peptide antigen	-26.3
-	-	GO:0019886	antigen processing and presentation of exogenous peptide antigen *via* MHC class II	-25.9
-	MCODE_1	GO:0019886	antigen processing and presentation of exogenous peptide antigen *via* MHC class II	-36.9
-	MCODE_1	GO:0002495	antigen processing and presentation of peptide antigen *via* MHC class II	-36.5
-	MCODE_1	GO:0002504	antigen processing and presentation of peptide or polysaccharide antigen *via* MHC class II	-36.4
-	MCODE_2	CORUM:6418	C1q complex	-12.1
-	MCODE_2	R-HSA-173623	Classical antibody-mediated complement activation	-10.8
-	MCODE_2	GO:0098883	synapse pruning	-10
-	MCODE_3	R-HSA-373755	Semaphorin interactions	-8
-	MCODE_3	GO:0009611	response to wounding	-5.6
-	MCODE_3	GO:0010720	positive regulation of cell development	-5.5
-	MCODE_4	R-HSA-194315	Signaling by Rho GTPases	-4.9
-	MCODE_4	R-HSA-9716542	Signaling by Rho GTPases, Miro GTPases and RHOBTB3	-4.9
-	MCODE_5	hsa04380	Osteoclast differentiation	-7.1
-	MCODE_5	GO:0002250	adaptive immune response	-5
IGLV2-14	Top3	-	-	-
-	-	GO:0002252	immune effector process	-14.9
-	-	GO:0002250	adaptive immune response	-13.9
-	-	GO:0002460	adaptive immune response based on somatic recombination of immune receptors built from immunoglobulin superfamily domains	-13.9
-	MCODE_1	GO:0002250	adaptive immune response	-4
-	MCODE_1	R-HSA-9824446	Viral Infection Pathways	-3.8
-	MCODE_1	R-HSA-1280218	Adaptive Immune System	-3.8
-	MCODE_2	GO:0072678	T cell migration	-8.9
-	MCODE_2	GO:0072676	lymphocyte migration	-7.8
-	MCODE_2	GO:0071674	mononuclear cell migration	-7.3
HLA-DQA1	Top3		-	-
-	-	hsa05150	Staphylococcus aureus infection	-27.5
-	-	WP2328	Allograft rejection	-25.9
-	-	hsa04145	Phagosome	-25.8
-	MCODE_1	GO:0019886	antigen processing and presentation of exogenous peptide antigen *via* MHC class II	-31.2
-	MCODE_1	GO:0002495	antigen processing and presentation of peptide antigen *via* MHC class II	-30.8
-	MCODE_1	GO:0002504	antigen processing and presentation of peptide or polysaccharide antigen *via* MHC class II	-30.7
-	MCODE_2	R-HSA-375276	Peptide ligand-binding receptors	-8.7
-	MCODE_2	R-HSA-418594	G alpha (i) signaling events	-7.9
-	MCODE_2	R-HSA-373076	Class A/1 (Rhodopsin-like receptors)	-7.8
-	MCODE_3	CORUM:6418	C1q complex	-12.1
-	MCODE_3	GO:0006958	complement activation, classical pathway	-11.6
-	MCODE_3	GO:0002455	humoral immune response mediated by circulating immunoglobulin	-11.4
-	MCODE_5	GO:0002445	type II hypersensitivity	-10.9
-	MCODE_5	GO:0001788	antibody-dependent cellular cytotoxicity	-10.9
-	MCODE_5	GO:0001794	type IIa hypersensitivity	-10.9

**Table 4:** Core gene PPI of positive myeloid dendritic cells for each causal relationship gene.

**Gene Name**	**Network/MCODE**	**GO**	**Description**	**Log10(P)**
CD1C	Top3	-	-	-
-	-	GO:0019884	Antigen processing and presentation of exogenous antigen	-33.1
-	-	GO:0019886	Antigen processing and presentation of exogenous peptide antigen *via* MHC class II	-32.1
-	-	GO:0002495	Antigen processing and presentation of peptide antigen *via* MHC class II	-31.6
-	MCODE_1	GO:0019886	Antigen processing and presentation of exogenous peptide antigen *via* MHC class II	-35.8
-	MCODE_1	GO:0002495	Antigen processing and presentation of peptide antigen *via* MHC class II	-35.4
-	MCODE_1	GO:0002504	Antigen processing and presentation of peptide or polysaccharide antigen *via* MHC class II	-35.2
-	MCODE_2	hsa05130	Pathogenic Escherichia coli infection	-5.6
-	MCODE_2	hsa05132	Salmonella infection	-5.3
-	MCODE_3	hsa05323	Rheumatoid arthritis	-12.6
-	MCODE_3	hsa05417	Lipid and atherosclerosis	-10.8
-	MCODE_3	R-HSA-6783783	Interleukin-10 signaling	-10.6
-	MCODE_4	GO:0038093	Fc receptor signaling pathway	-8.3
-	MCODE_4	M7	PID FCER1 PATHWAY	-8.1
-	MCODE_4	GO:0016064	Immunoglobulin mediated immune response	-6.8
HLA-DRA1	Top3	-	-	-
-	-	GO:0050778	Positive regulation of immune response	-37.3
-	-	GO:0006954	Inflammatory response	-36.3
-	-	R-HSA-6798695	Neutrophil degranulation	-35.4
-	MCODE_1	hsa04612	Antigen processing and presentation	-34.7
-	MCODE_1	GO:0019886	Antigen processing and presentation of exogenous peptide antigen *via* MHC class II	-33.7
-	MCODE_1	GO:0002495	Antigen processing and presentation of peptide antigen *via* MHC class II	-33.3
-	MCODE_2	M159	PID AMB2 NEUTROPHILS PATHWAY	-11.6
-	MCODE_2	M174	PID UPA UPAR PATHWAY	-11.5
-	MCODE_2	GO:0006954	Inflammatory response	-11.2
-	MCODE_3	hsa04670	Leukocyte transendothelial migration	-9.7
-	MCODE_3	R-HSA-1236973	Cross-presentation of particulate exogenous antigens (phagosomes)	-8.8
-	MCODE_3	R-HSA-4420097	VEGFA-VEGFR2 Pathway	-7.7
-	MCODE_4	R-HSA-418594	G alpha (i) signaling events	-15.9
-	MCODE_4	R-HSA-388396	GPCR downstream signaling	-13.5
-	MCODE_4	R-HSA-372790	Signaling by GPCR	-13.1
-	MCODE_5	hsa05208	Chemical carcinogenesis - reactive oxygen species	-5.1
-	MCODE_5	GO:2001234	Negative regulation of apoptotic signaling pathway	-5
-	MCODE_5	hsa05020	Prion disease	-4.9
-	MCODE_6	hsa04380	Osteoclast differentiation	-14.2
-	MCODE_6	GO:0032680	Regulation of tumor necrosis factor production	-13.3
-	MCODE_6	GO:1903555	Regulation of tumor necrosis factor superfamily cytokine production	-13.2
-	MCODE_7	R-HSA-166786	Creation of C4 and C2 activators	-13.5
-	MCODE_7	R-HSA-166663	Initial triggering of complement	-12.6
-	MCODE_7	CORUM:6418	C1q complex	-12.1
-	MCODE_8	GO:0030162	Regulation of proteolysis	-4.5
-	MCODE_8	GO:0031329	Regulation of cellular catabolic process	-4.2
HLA-DRB1	Top3	-	-	-
-	-	GO:0050778	Positive regulation of immune response	-33.2
-	-	GO:0019886	Antigen processing and presentation of exogenous peptide antigen *via* MHC class II	-28
-	-	GO:0002495	Antigen processing and presentation of peptide antigen *via* MHC class II	-27.4
-	MCODE_1	GO:0019886	Antigen processing and presentation of exogenous peptide antigen *via* MHC class II	-35.8
-	MCODE_1	GO:0002495	Antigen processing and presentation of peptide antigen *via* MHC class II	-35.4
-	MCODE_1	GO:0002504	Antigen processing and presentation of peptide or polysaccharide antigen *via* MHC class II	-35.2
-	MCODE_2	M169	PID INTEGRIN2 PATHWAY	-9.7
-	MCODE_2	R-HSA-977225	Amyloid fiber formation	-7.3
-	MCODE_2	M65	PID FRA PATHWAY	-6.6
-	MCODE_3	R-HSA-6783783	Interleukin-10 signaling	-17.8
-	MCODE_3	GO:0006954	Inflammatory response	-14.6
-	MCODE_3	R-HSA-373076	Class A/1 (Rhodopsin-like receptors)	-14
-	MCODE_5	R-HSA-2730905	Role of LAT2/NTAL/LAB on calcium mobilization	-9.9
-	MCODE_5	M7	PID FCER1 PATHWAY	-8.1
-	MCODE_5	GO:0002275	Myeloid cell activation involved in immune response	-8

## Data Availability

All data generated or analyzed during this study are included in this published article.

## LIST OF ABBREVIATIONS

HEIDI: Heterogeneity in Dependent Instruments
IRB: Ethical Review Board
PPI: Protein-protein Interaction
SMR: Summary-data-based Mendelian Randomization
