# Supplementary material for: Integrative Analysis Reveals Genes Causal Relation with Ovarian Cancer and aging
Source: Curr Top Med Chem. 2025 May 9;26(7):721–46. doi: 10.2174/0115680266358687250507041056 (PMC13478936; doi:10.2174/0115680266358687250507041056)
Supplement: Supplementary file 1 [file CTMC-26-7-721_SD1.pdf]

Supplementary Material

Integrative Analysis Reveals Genes Causal Relation with Ovarian Cancer and aging

Lan-hui Qin[1,2,#], Chongze Yang[1,#] , Rui Song[1], Pei-yin Chen[2], Zijian Jiang[1], Weihui Xu[2], Guanzhen Zeng[2], Jin-yuan Liao[2,*] and Liling Long[2,*]

[1]Department of Radiology, Guangxi Medical University, No.22 Shuangyong Road, Nanning 530021, Guangxi, China; [2]Department of Radiology, First Affiliated Hospital of Guangxi Medical University, No.6 Shuangyong Road, Nanning, Guangxi 530021, China

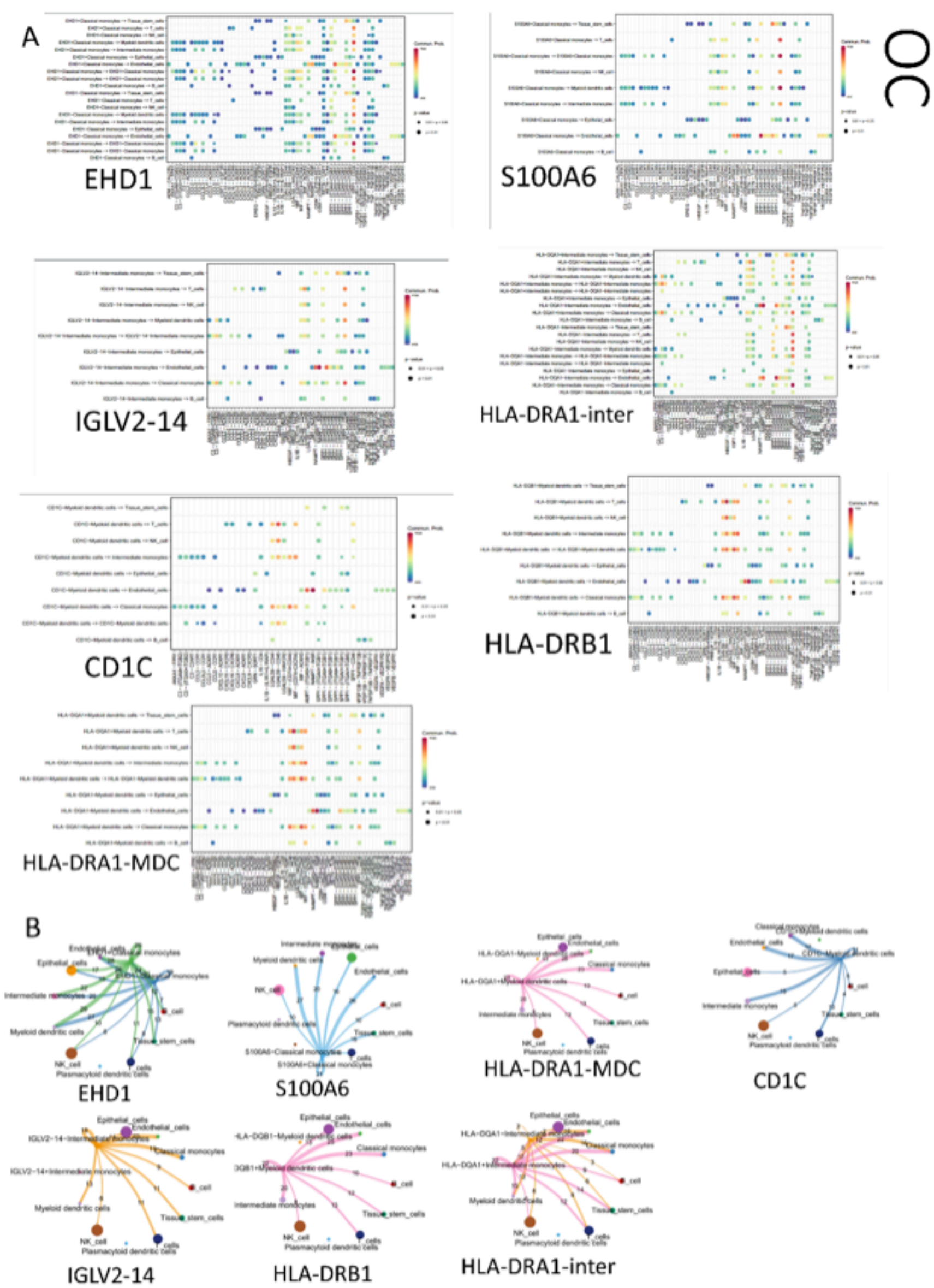

S Fig. (1). Cell communication analysis of causal relationship gene-positive monocytes in ovarian cancer. A) Ligand-receptor pairs of causal relationship gene-positive monocytes in ovarian cancer; B) Cell interaction analysis of causal relationship gene-positive monocytes in ovarian cancer.

OC, ovarian cancer

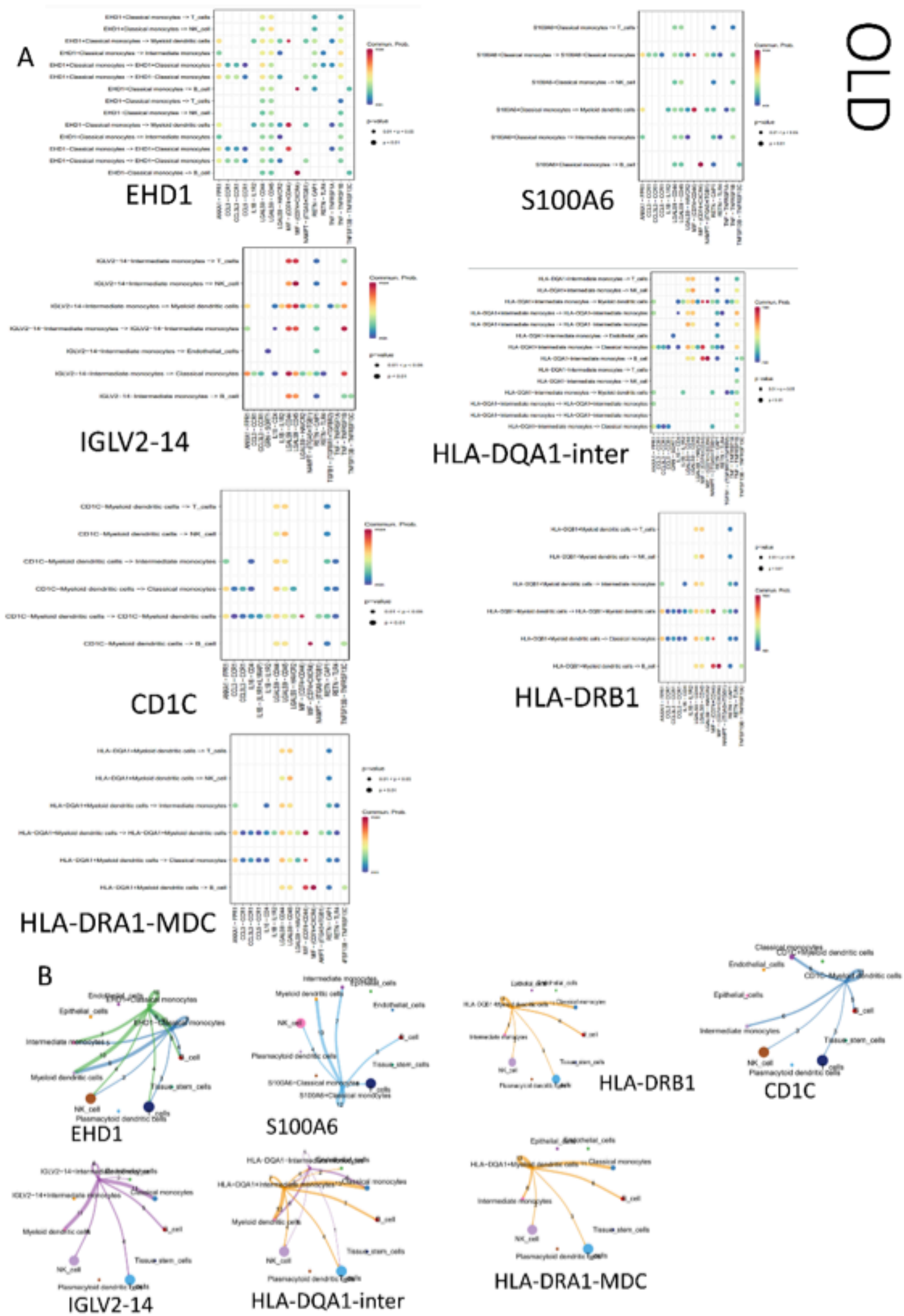

S Fig. (2). Cell communication analysis of causal relationship gene-positive monocytes in aging. A) Ligand-receptor pairs of causal relationship gene-positive monocytes in aging; B) Cell interaction analysis of causal relationship gene-positive monocytes in aging.

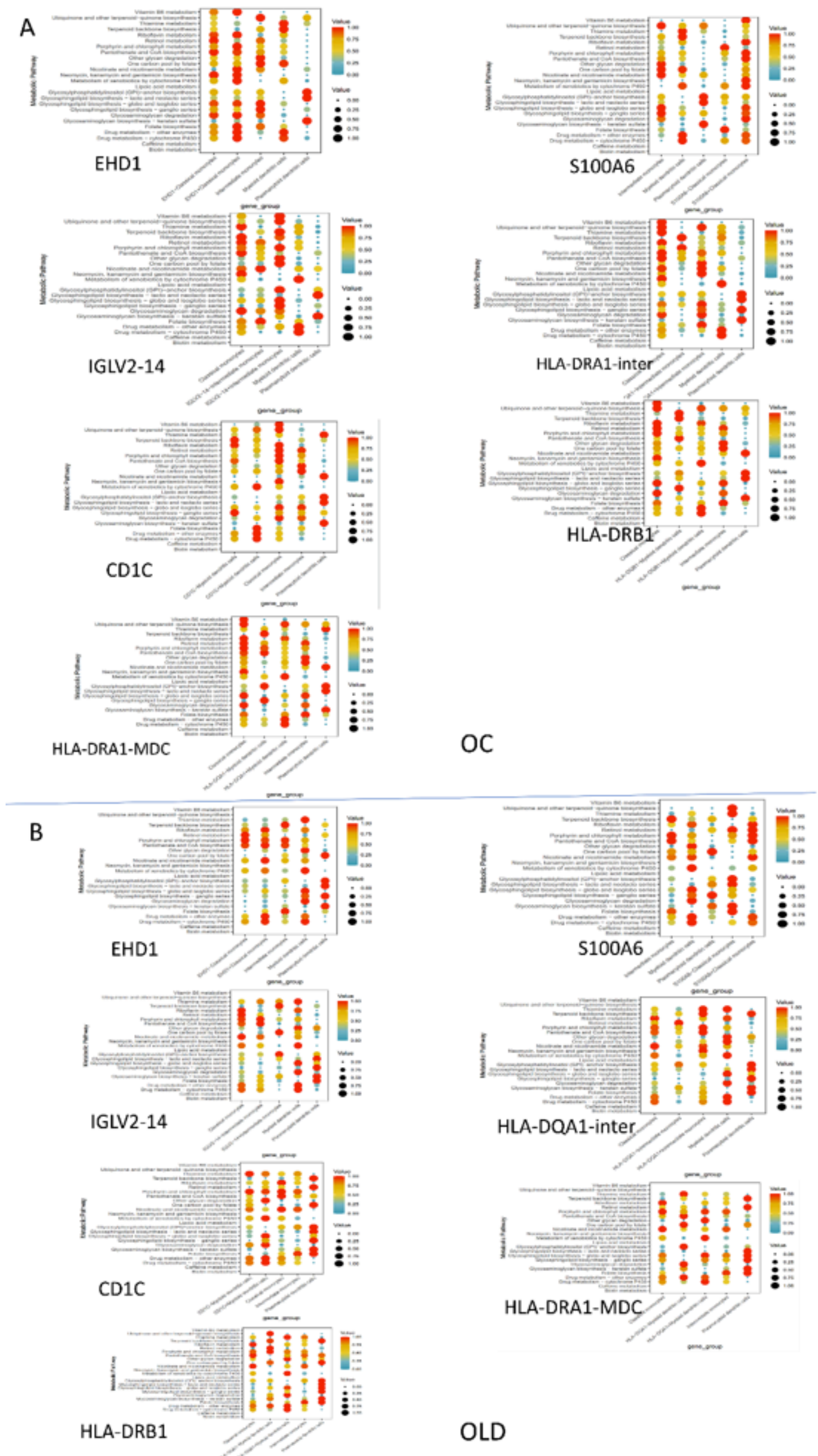

S Fig. (3). Metabolic pathways of causal relationship gene-positive monocytes in ovarian cancer and aging. A) Metabolic pathways of causal relationship gene-positive monocytes in ovarian cancer (OC). B) Metabolic pathways of causal relationship gene-positive monocytes in aging.

OC ovarian cancer

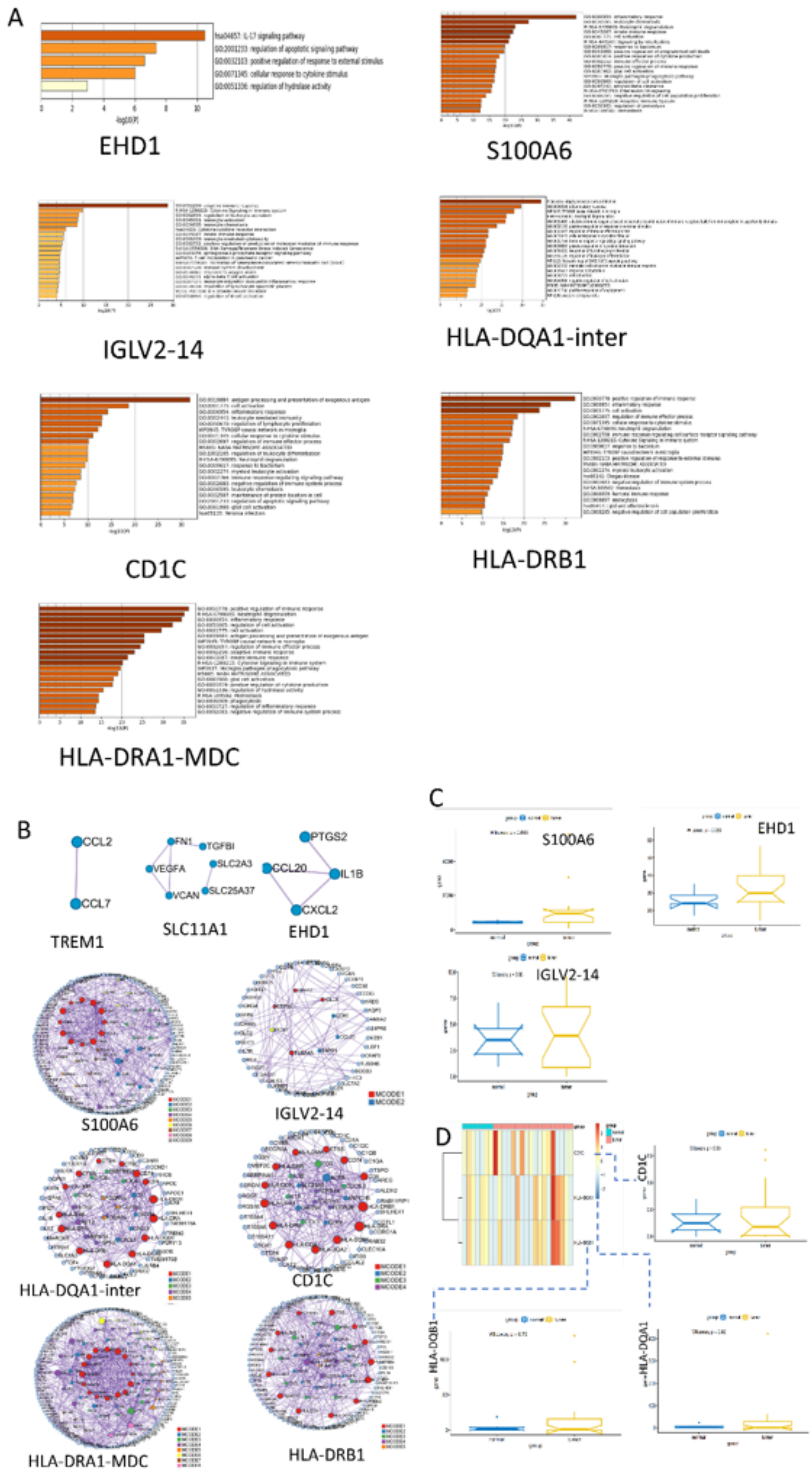

S Fig. (4). Analysis of core gene enrichment pathways, PPI, and validation of causal relationship genes in monocytes positive for causal relationship genes in ovarian cancer and aging. A) Core gene enrichment pathways in monocytes positive for causal relationship genes; B) Core gene PPI (Protein-Protein Interaction) in monocytes positive for causal relationship genes; C) Validation analysis of causal relationship genes in monocyte subtypes.
